# Breast and Formula Milk and Early Puberty Onset

**DOI:** 10.3390/children10101686

**Published:** 2023-10-14

**Authors:** Valeria Calcaterra, Hellas Cena, Francesca Sottotetti, Virginia Rossi, Federica Loperfido, Gianvincenzo Zuccotti

**Affiliations:** 1 Department of Internal Medicine and Therapeutics, University of Pavia, 27100 Pavia, Italy; valeria.calcaterra@unipv.it; 2 Pediatric Department, Buzzi Children’s Hospital, 20154 Milano, Italy; virginia.rossi@unimi.it (V.R.); gianvincenzo.zuccotti@unimi.it (G.Z.); 3 Laboratory of Dietetics and Clinical Nutrition, Department of Public Health, Experimental and Forensic Medicine, University of Pavia, 27100 Pavia, Italy; francesca.sottotetti@unipv.it (F.S.); federica.loperfido@unipv.it (F.L.); 4 Clinical Nutrition Unit, General Medicine, Istituti Clinici Scientifici Maugeri IRCCS, 27100 Pavia, Italy; 5 Department of Biomedical and Clinical Science, University of Milano, 20157 Milano, Italy

**Keywords:** breastfeeding, formula feeding, infant formula, milk, puberty, early puberty onset, precocious puberty

## Abstract

Nutrients have an enormous impact on many hormonal systems and aspects of health, and nutrition status is a crucial regulator of growth and pubertal development in children and adolescents. In this narrative review, we explore the connection between these feeding methods and the timing of puberty to provide a clearer understanding of how infant nutrition might contribute to the early development of puberty. Puberty is a key stage in the transition from childhood to adulthood and the timing of puberty represents a significant biological milestone of growth. Breast milk seems to have a pivotal role in puberty onset, mainly due to its dynamism, which shape indirectly the gut microbiota in early life, besides direct exposure of the baby to the milk microbiota through gut–breast axis. Concerning breast and formula milk and their effects on the onset of puberty, a protective role of the former occurs. As for the potential harmful effects of soy-based formulas and the isoflavones that they contain, the studies reported demonstrate conflicting opinions, underlining the need for further research on this topic. A healthy and well-nourished diet from the earliest stages of life has significant preventive potential for overall well-being, reducing the risk of many health problems later in life.

## 1. Introduction

Nutrients have an enormous impact on many hormonal systems and aspects of health and nutrition status is a crucial regulator of growth and pubertal development in children and adolescents [1,2,3].

Puberty is a key stage in the transition from childhood to adulthood and the timing of puberty represents a significant biological milestone of growth [4,5]. Puberty timing is influenced by a range of factors, including nutrition, genetics, body mass index (BMI), endocrine disrupting chemicals [6]. Recent studies have sparked interest in investigating the impact of early childhood feeding practices, particularly breast milk and formula milk, on the onset of puberty.

Regarding breastfeeding, the World Health Organization (WHO) recommends it at least up to 6 months because of the positive impact on both mother and newborn health [7,8]. It is in fact known that breastfed infants have a lower risk of incurring respiratory, allergic, gastroenterological and endocrinological pathologies, compared to non-breastfed ones [9] However, since breastfeeding might not always be possible, infant formulae (IF) have been developed over the years and companies attempt to make it as similar as possible to breast milk, even if the latter presents a unique composition that is difficult to reproduce [10].

In this narrative review, we explore the characteristics of breast milk and formula milk and the connection between these feeding methods and the timing of puberty to provide a clearer understanding of how infant nutrition might contribute to the early development of puberty. This review seeks to shed light on the practical implications of early life nutrition in shaping pubertal patterns, considering both physiological and social aspects of this intricate relationship.

## 2. Methods

**Figure 1:**
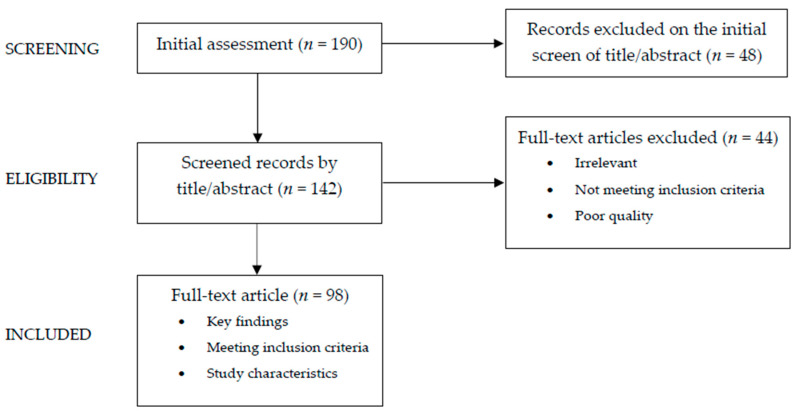
Diagram graphically showing the process of paper selection and exclusion used in writing this narrative review.

To conduct the narrative review, specific inclusion criteria were established: articles written in English, meta-analyses, clinical trials, and reviews focusing on the subject within the last twenty years were considered. Case reports and series were excluded. The review utilized the electronic databases PubMed, Scopus, and Web of Science. Relevant keywords were used either individually or in combination. The key words used for this research alone or/and in association were breastfeeding, formula feeding, infant formula, milk, puberty, early puberty onset, and precocious puberty. The time period for conducting keyword search was 2003–2023. Starting with a total of 190 papers, the authors independently assessed abstracts (n = 142) and then examined full texts to identify potentially relevant studies (n = 98) from the literature (Figure 1). Additionally, the reference lists of all articles were reviewed to find pertinent studies.

## 3. Breast Milk and Early Puberty Onset

Nutrition plays a pivotal role in the development of puberty [1]. The connection between nutrition and the puberty onset is closely linked to the relationship between the amount of food intake and weight gain pattern, sometimes leading to obesity [2,3]. Early life nutrition, before two years of age, splits into breastfeeding or formula feeding and, later, complementary foods [6].

Breast milk is the ideal initial food for newborns, with recommendations from the WHO) [8], the Academy of Nutrition and Dietetics [11] and the American Academy of Pediatrics (AAP) supporting exclusive breastfeeding for at least the first six months [12]. This recommendation is aligned with guidelines from the American College of Obstetricians and Gynecologists and the Canadian Pediatric Society [13,14]. Furthermore, the Academy of Nutrition and Dietetics recommends associating breastfeeding with the gradual introduction of complementary foods until 12 months of age [11] and the AAP advocates continuing breastfeeding, along with appropriate complementary foods introduced at about 6 months, as long as the mother and baby mutually desire it, for 2 years or more [12].

Early life nutrition continues to have a lasting positive impact on health well into adulthood. Numerous data confirm that breastfed infants experience lower occurrences of various acute and chronic pediatric disorders, such as respiratory, allergologic, gastroenterologic, and endocrinologic conditions, as compared to non-breastfed infants [9]. This beneficial effect may be attributed to the unique biological composition of human milk. Additionally, breastfeeding mothers themselves enjoy a reduced risk of developing type 2 diabetes mellitus, as well as breast, ovarian, and endometrial cancer, and hypertension [9].

Different studies have examined the influence of breastfeeding on pubertal development, but the findings are not always consistent.

Breastfeeding’s influence on the timing of puberty appears to yield varying outcomes based on several studies. Hvidt et al.’s population-based cohort study [15], focusing on 13,511 boys and girls from the Puberty Cohort nested within the Danish National Birth Cohort, found intriguing gender disparities. In boys, the absence of breastfeeding was linked to accelerated pubertal timing. However, for girls, breastfeeding did not show a significant association with pubertal development. Similarly, Al-Sahab et al. [16] found in their population-based cohort study, drawing data from the Cebu Longitudinal Health and Nutrition Survey, that exclusive breastfeeding was negatively associated with early menarche. In contrast, Karaolis-Danckert et al.’s cohort study [17] conducted in Germany suggested that intrauterine and early postnatal growth factors, including full breastfeeding for at least 4 months, influenced markers of puberty onset in both boys and girls independently of prepubertal body composition. Aghaee et al. [18], in their prospective cohort study involving a multiethnic cohort of mother–daughter pairs, reported that the absence of breastfeeding was linked to earlier onset of breast and pubic hair development in girls compared to those breastfed for at least 6 months. Kale et al. [19], in their prospective study encompassing diverse girls across geographic locations, found mixed effects. Mainly breastfed or mixed-fed girls had a delayed breast development onset compared to formula-fed peers, while breastfeeding’s impact on pubic hair development onset was not significant. In contrast, Kwok et al.’s observational cohort study [20] involving the “Children of 1997” cohort from Hong Kong indicated that neither breastfeeding nor childhood milk consumption had a connection with the timing of pubertal onset. Morris et al.’s cohort study [21] in the UK’s Breast Cancer Generations Study showed that breastfeeding correlated with a delayed onset of menarche in women. The Thousand Families Study by Blell et al. [22], a prospective longitudinal cohort study in the UK, revealed that the timing of menarche was not associated with the duration of breastfeeding. On the other hand, Ong et al. [23] demonstrated in the Avon Longitudinal Study of Parents and Children (ALSPAC) cohort that breast milk might act protectively against earlier menarche in girls. Lee et al. [24], in their prospective observational study in South Korea, found that children breastfed for 6 months or more exhibited a protective link against early puberty.

Overall, these studies highlight the complex and sometimes inconsistent role of breastfeeding in influencing the timing of puberty. The outcomes appear to be influenced by factors such as gender, duration of breastfeeding, and other contextual variables, underlining the need for further investigation to better understand these relationships. Table 1 summarizes the results of these studies, selected for their significance.

A plausible link between early life nutrition, breastfeeding duration (if applicable), and the timing of puberty has been elucidated, particularly in relation to the correlation between overweight/obesity and the risk of early puberty. Breastfeeding during the first year of life is a notable factor implied in a reduction of childhood overweight likelihood by 15% in comparison to formula feeding [25]. This protective attribute is linked to divergent growth patterns between breastfed and formula-fed infants [6,26,27]. Breastfed babies typically follow a slower growth curve, a characteristic that appears to safeguard against later-life obesity [6,28,29]. Even after accounting for birth weight, the correlation between rapid weight gain, known as ‘catch-up growth’, and obesity retains significance [30].

The linkage between early life nutrition and the timing of puberty draws from the association between increased weight gain during early childhood and higher childhood BMI, both of which have been linked to an earlier onset of puberty [3,31]. This correlation finds support in the interconnected factors that contribute to the relationship between breast milk and the prevention of excessive weight gain and early puberty. These elements encompass insulin-like growth factor (IGF)-1 levels, leptin levels, bioactive nutrients present in both human and formula milk, the composition of the microbiome, and the aptitude for self-regulated feeding [6,32,33,34]. Elevated IGF-1 levels manifest both in formula-fed infants [32] and infants experiencing swift weight gain in early months [35]. Importantly, increased IGF-1 levels correlate with enhanced sex steroid production, which facilitates secretion of GnRH and subsequent pubertal development [36].

Rapid weight gain during infancy, as shown by Emmett PM et al. [37], associates with increased risk of obesity at five and eight years, coupled with heightened insulin resistance, advanced adrenarche, and reduced sex hormone-binding globulin levels. Notably, Zheng [30] highlighted substantial connections between swift weight gain and obesity, even after birth weight adjustment, thereby suggesting an influence on puberty timing. Li et al. [38] confirmed that breastfeeding was inversely associated with the risk of childhood obesity from 2 to 6 years old, and there was a trend from mixed feeding to exclusive breastfeeding; infant exclusive formula feeding might be a risk factor for childhood underweight at preschool time [38].

Across various nonhuman species, the timing of puberty is influenced by the social environment, carrying implications for reproductive success and behavioral patterns [39]. Within the realm of human beings, although variations in pubertal timing have not yet been systematically investigated concerning social environmental factors, it is noteworthy that their psychological consequences have been documented.

For instance, the quality of child–parent attachment has shown a positive correlation with breastfeeding, and it appears to exert an influence on reproductive development during later stages of life [40,41]. Conversely, maternal depression, which exhibits a negative association with breastfeeding, has been linked to an increased likelihood of early pubertal onset in girls [5,6,42].

## 4. Formula Milk and Early Puberty Onset

As we already underlined, over the years, it has been studied how early childhood nutrition can influence the pubertal process [5]. Specifically, attention is increasingly directed to the difference between breast milk and formula milk and the effects that these may have on pubertal development [19]. In a study conducted by Novotny R. et al., a sample of girls residing on the island of Oahu was evaluated based on their feeding during early childhood; of the 349 girls enrolled, 59 (17%) were breastfed only, while 242 (71%) were formula fed. The authors concluded that the formula-fed girls were shorter and heavier than the breast-fed ones, resulting in early attainment of menarche [43]. In 2012, a study compared mental, motor, and language development between breastfed infants and infants fed with milk-based formula or soy protein-based formula during the first year of life. The results showed that there were no significant differences between the infants fed with the two types of formulas. However, breastfed infants showed an improved cognitive and psychomotor development compared to formula-fed infants [44]. A prospective cohort investigation revealed that among a sample of 1237 girls aged 6 to 8 years, those mainly nourished through breastfeeding experienced a delayed initiation of breast development compared to those who were formula fed. Moreover, breastfeeding duration was also directly associated with age at the onset breast initiation development [19]. Finally, a randomized controlled trial conducted in 2018 compared a significant group of infants fed cow’s milk formula (CMF) with children who received extensively hydrolyzed formula (EHF). There was a significant difference in the rate of weight gain velocity from 0.75 to 4.5 months (*p* = 0.002) between the two groups being compared. Specifically, the EHF group gained weight on average by 25.1 ± 0.9 g/d, while the CMF group by 29.0 ± 1.0 g/day. However, there was no difference in length increase [45]. In this scenario, opinions about the use of soy-based formulas are also conflicting, due to the presence of high concentrations of isoflavones and its potential estrogenic effect on infant development [6]. Isoflavones constitute a cluster of phytoestrogens encompassing three primary compounds (genistein, daidzein, and glycitein) with a chemical composition and hormonal effects similar to estradiol [46]. Indeed, a study involving 2920 girls has observed that about 2% had started introducing soy-based formula at or before the age of 4 months. The study’s overall findings revealed that the median age at which the girls experienced menarche was around 12.8 years. However, the girls fed with soy-based formulae experienced menarche by 4 months earlier (12.4 years). Moreover, the girls who consumed soy products had a 25% higher risk of experiencing early onset menarche than girls who were fed formula or non-soy milk (Hazard Ratio of 1.25 (95% confidence interval, 0.92, 1.71)). The authors concluded that exposure to soy isoflavones may have mild endocrine-disrupting effects, potentially leading to the early onset of puberty. Nevertheless, they highlighted that their study had some limitations, primarily due to the relatively small sample size [47]. Otherwise, Sinai et al. found no significant differences in pubertal development, growth, or BMI values in a nested case–control study in which they examined the possible association between soy formula feeding and early pubertal manifestation [48]. More recently, another case–control study demonstrated that exclusive breastfeeding up to 6 months of age was an important protective factor for CPP; instead, soy consumption was positively correlated (r = 0.2; *p* < 0.01) [46]. Thus, although the association between early soy exposure and early menarche is biologically plausible, different studies have produced conflicting results and further research is needed to elucidate this topic. Table 2 summarizes the papers referring to formula milk and early puberty onset.

## 5. Conclusions

Nutrition plays a central role in human growth and development, particularly in relation to childhood obesity and the long-term impact of early life nutrition.

Breast milk is a unique source of beneficial bacteria and compounds that shape an infant’s microbiota and influence various bodily functions’ development, such as gastrointestinal, immunological, and neurological.

The timing of puberty is a significant milestone in growth, influenced by early nutrition and breastfeeding, which is associated with lower rates of pediatric disorders. The HPG axis plays a key role in regulating puberty onset, and breast milk’s dynamic composition indirectly affects the gut microbiota, further influencing this process.

Breast milk is believed to have a protective role in puberty onset, while soy-based formulas containing isoflavones have conflicting research findings regarding their potential harmful effects, which require further investigation.

This manuscript, being a narrative review, takes a less formal methodologic approach than systematic reviews, but provides an analysis of how feeding practices during the first stages of life may contribute to infants’ growth processes, including pubertal patterns.

Overall, it emphasizes the importance of a well-nourished diet in early childhood that promotes healthy growth, prevents chronic diseases and supports mental well-being. Establishing healthy dietary habits early in life is considered a key tool for improving long-term health and reducing the burden of chronic disease for both individuals and health systems.

## Figures and Tables

**Table 1:** Summary of the papers referred to breastfeeding and early puberty onset.

Author, Year	Study Design	Cohort	Results
Hvidt et al., 2021 [15]	Population-based cohort study	In total, 13,511 boys and girls from the Puberty Cohort nested within the Danish National Birth Cohort.	YES. Absence of breastfeeding accelerate pubertal timing in boys; in girls, breastfeeding was not associated with pubertal development.
Al-Sahab et al., 2011 [16]	Population-based cohort study	Data was taken from the Cebu Longitudinal Health and Nutrition Survey, initiated in 1981 to examine the feeding patterns of infants in the Metro Cebu Area, Philippines, recruiting pregnant women who gave birth between May 1983 and April 1984. Follow-up studies were conducted when the girls reached ages 8–9, 11–12, and 14–15 years.	NO. Negative association between exclusive breastfeeding and early menarche.
Karaolis-Danckert et al., 2009 [17]	Cohort study conducted by the Research Institute of Child Nutrition in Dortmund, Germany	German girls and boys between 6 and 13 years. Only term (37–42 wk of gestation) singletons with a birth weight 2500 g who fulfilled the following minimum requirement were selected.	YES. In both boys and girls, intrauterine and early postnatal growth factors (including full breastfeeding, >= 4 months) appear to influence both early and later markers of puberty onset independently of prepubertal body composition.
Aghaee et al., 2019 [18]	Prospective cohort	This study considered a multiethnic cohort, eventually including 3331 mother–daughter pairs (girls born between 2004 and 2006).	YES. Absence of breastfeeding has been linked to an earlier onset of breast and pubic hair development, as compared to a breastfeeding period lasting at least 6 months.
Kale et al., 2015 [19]	Prospective study	This study considered a population of 1237 socio-economically and ethnically diverse girls across three geographic locations (New York City, Cincinnati, and the San Francisco Bay Area).	YES and NO. The effect of breastfeeding on pubertal onset varies by study site. Mainly breastfed or mixed-fed girls had a later onset of breast development compared to formula-fed peers; breastfeeding’s link to pubic hair development onset was not significant.
Kwok et al., 2012 [20]	Observational cohoty study	“Children of 1997” cohort from Hong Kong.	NO. Neither breastfeeding nor childhood milk consumption had a connection with the timing of pubertal onset.
Morris et al., 2010 [21]	Cohort study	The study cohort comprises women aged 16 and above from the UK who joined the Breast Cancer Generations Study. It started in 2003 with 111,595 participants. The final analysis included 81,606 women.	YES. Women who received breastfeeding experienced a delayed onset of menarche compared to those who were not breastfed.
Blell et al., 2008 [22]	Prospective longitudinal cohort study	The cohort in the Thousand Families Study includes all 1142 children who were born in May and June 1947 to mothers residing in Newcastle-upon-Tyne, UK. Out of the original cohort, a total of 832 individuals were successfully traced.	NO. Timing of menarche was not associated with the duration of breast-feeding.
Ong et al., 2009 [23]	Prospective cohort study	The cohort of the Avon Longitudinal Study of Parents and Children (ALSPAC) study is formed by 14,541 pregnant women who resided in one of three Bristol-based health districts in the former County of Avon.	YES. Breast milk appears to be protective against earlier menarche in girls.
Lee et al., 2015 [24]	Prospective observational study	The study involves the Ewha Birth & Growth Cohort (2001–2006, South Korea). It follows mothers and their children, with clinical assessments performed at ages 3, 5, 7, 8, and 9. The 2011 follow-up included 260 participants aged 9, focusing on puberty.	YES. Children breastfed for 6 months or more exhibited a protective link against early puberty

**Table 2:** Summary of the papers referred to formula milk and early puberty onset.

Author, Year	Study Design	Cohort	Results
Novotny R. et al., 2003 [43]	Comparative study	Girls residing on the island of Oahu were evaluated based on their feeding during early childhood; of the 349 girls enrolled, 59 (17%) were breastfed only, while 242 (71%) were formula fed.	YES Formula-fed girls exhibited shorter stature and greater weight compared to the breastfed ones, leading to an earlier onset of menarche.
Andres A. et al., 2012 [44]	Clinical trial	Overall, 391 Healthy infants were divided in 3 groups: breastfed (BF), milk-based formula-fed (MF), or soy protein-based formula-fed (SF). Development was evaluated (at ages 3, 6, 9, and 12 months) by using the Bayley Scales of Infant Development and the Preschool Language Scale-3.	NO No significant differences between the infants fed with the two types of formulas. YES Breastfed infants showed an improved cognitive and psychomotor development than formula-fed infants.
Kale A. et al., 2015 [19]	Prospective cohort study	Overall, 1237 girls (age between 6 and 8) were enrolled from three different regions: New York City, Cincinnati, and the San Francisco Bay Area. The study evaluated breastfeeding habits through self-administered questionnaires or interviews conducted with the caregiver.	YES Girls who were predominantly breastfed had a later onset of breast development compared with those who were formula-fed. Breastfeeding duration was also directly associated with age at the onset of breast development.
Mennella JA et al., 2018 [45]	Randomized controlled trial	A comparison between a significant group of infants fed cow’s milk formula (CMF) with children who received extensively hydrolysed formula (EHF).	YES Significant difference in the rate of weight gain velocity from 0.75 to 4.5 months (*p* = 0.002) between the two groups being compared. Specifically, the EHF group gained weight on average by 25.1 ± 0.9 g/d, while the CMF group by 29.0 ± 1.0 g/day. No difference in length increase.
Adgent MA et al., 2012 [47]	Prospective, longitudinal study	A sample of 2920 girls; approximately 2% had introduced soy-based formula at 4 months of age or earlier.	YES Girls fed soy products had a 25% higher risk of developing premature menarche than girls fed formula or non-soy milk (Hazard Ratio 1.25 (95% confidence interval, 0.92, 1.71)). The presence of mild endocrine disrupting effects of soy isoflavone exposure, may lead to an early onset of puberty. However, the research was limited to a few subjects exposed to soy
Sinai et al., 2019 [48]	Nested case–control study	A cohort of children was followed prospectively from birth to age 3; during this period, their eating habits and the possible appearance of IgE-mediated cow’s milk allergy were evaluated. Two groups were created: one included infants who consumed only soy-based infant formula (soy group: 29 participants, 17 males), the other group was randomly selected from those without IgE-CMA and who did not receive milk soy-based artificial (control group: 60 participants, 27 males).	NO No significant differences in pubertal development, growth, or BMI values.
Felício JS et al., 2021 [46]	Case-control study	The study involved 161 girls, of which 84 were patients diagnosed with CPP (case group), the remaining 77 did not have a diagnosis of CPP (control group).	YES Exclusive breastfeeding up to 6 months of age, was an important protective factor for CPP; instead, soy consumption was positively correlated (r = 0.2; *p* < 0.01).

## Data Availability

Not applicable.
